# Woody vegetation structure of relict *Abies jaliscana* forests in western Mexico: characteristics, environmental drivers and conservation implications

**DOI:** 10.7717/peerj.21342

**Published:** 2026-06-12

**Authors:** Ricardo Guerrero-Hernández, Gerardo Hernández-Vera, Miguel Angel Muñiz-Castro, Ramón Cuevas-Guzmán

**Affiliations:** 1 Centro Interdisciplinario de Investigación para el Desarrollo Integral Regional, Unidad Durango. Sistemática y Ecología Vegetal, Instituto Politécnico Nacional, Durango, Durango, Mexico; 2 Centro Universitario de Ciencias Biológicas y Agropecuarias. Departamento de Botánica y Zoología, Universidad de Guadalajara, Zapopan, Jalisco, Mexico; 3 Centro Universitario de la Costa Sur. Instituto Manantlán de Ecología y Conservación de la Biodiversidad, Universidad de Guadalajara, Autlán de Navarro, Jalisco, Mexico

**Keywords:** Fir Forest, Biodiversity Conservation, Endangered Species, Vegetation Structure, *Abies*, Plant Communities

## Abstract

*Abies jaliscana* (Jalisco fir) is an endemic fir species limited to six populations in a transition zone between the Trans-Mexican Volcanic Belt (TMVB) and Sierra Madre del Sur (SMS) mountain range, in Western Jalisco, Mexico. Despite its relictual distribution, these populations and forests are continually threatened, and studies focusing on microenvironmental and climatic factors affecting forest structure are much needed for their effective management and conservation. Here we assess vegetation stand characteristics, current distribution, and the relationships among climate and microenvironmental factors with vegetation structure in six localities that contain all the known relict populations of *A*. *jaliscana*. We sampled 38 study sites (plots) of 0.1 ha across a latitudinal geographic range of ca. 100 km, and woody species were identified and measured at each site. Sixteen environmental variables were recorded at each site, in four subsets: physiographical, disturbance, biotic and climatic.To explore relationships amongclimatic variables, microenvironmental factors and vegetation structure (fir trees and co-occurring species), we used non-metric multidimensional scaling (NMDS). Woody species ranged from three to 25 species per site in the canopy and the understorey. Basal area of fir trees varied between 21.65 and 68.89 m^2^/ha, and density from 130 to 495 stems/ha at the six localities. *A*. *jaliscana* comprised 18.2–98.7% of total basal area, and 2.11–76.32% of total density at each site, forming stands that ranged from nearly monospecific to codominant with *Quercus martinezii*, *Quercus obtusata*, *Pinus montezumae*, *Pinus pseudostrobus* and *Ternstroemia lineata*. Diameter distribution of Jalisco fir populations showed an inverted J-shape, a bell-shape and irregular patterns; the first one suggests a multiaged population, whereas the other two, indicate disturbance. NMDS results identified elevation and slope as the main variables associated with fir basal area. Basal area of fir trees is positively associated with gentle slopes at high elevation sites. The annual humidity index also contributed to explain differences in vegetation structure. Based on estimates of extent of occurrence and area of occupancy, and applying IUCN-Red List criteria we propose that *A*. *jaliscana* should be classified as an Endangered species. Our study provides evidence that climatic and microenvironmental conditions associated with elevation play an important role in determining the distribution limits and vegetation structure of relict temperate-like fir forests.

## Introduction

Persistence and dominance of temperate relict woody taxa in some tropical and subtropical montane ecosystems occur because these elements have been exposed to a sky-island dynamic in response to climate fluctuations (Mastretta-Yanes et al., 2015). However, other factors such as microenvironment, land-use history and biotic interactions (*e.g.*, interspecific competition) could also play a significant role on its presence and persistence. In Mexico, these relict boreal and temperate elements include *Abies*, *Acer*, *Fagus*, *Picea* and *Ulmus* (Graham, 1999); located in refugia (*i.e.,* habitats that operate on evolutionary time scales, offering better environmental conditions to many species) which allow them to survive and persist, particularly under climate change (Keppel et al., 2012). The latter, over time, has had effects on the segregation of flora along elevational, latitudinal and topographic gradients. Recently, forest ecosystems in tropical latitudes have experienced more abrupt changes in their elevational distribution, unlike those in temperate latitudes (Colwell et al., 2008; Krishnaswamy, John & Joseph, 2014).

The genus *Abies* Mill. (fir) is one example of many Tertiary (Miocene-Pliocene) relict temperate taxa that have diversified into different species and now occur scattered at low latitudes. *Abies* includes ca. 50 species, mainly restricted to boreal and temperate latitudes but are also found in high mountains of subtropical and tropical regions (Mediterranean basin, Southeast Asia and highlands in Mexico-Guatemala) of the Northern Hemisphere (Aguirre-Planter et al., 2012; Semerikova & Semerikov, 2014). It is inferred that the most recent common ancestor of current Mesoamerican fir trees was part of the Arcto-Tertiary Flora, in the Northern Hemisphere during the late Cretaceous and Palaeogene, which was fragmented following climate cooling during the Neogene and Quaternary (Williams-Linera, Rowden & Newton, 2003; Deng et al., 2015). Phylogenetic and biogeographic studies, suggest an origin and early diversification of *Abies* in high-latitude mountains around the Pacific Ocean during the Eocene (Xiang et al., 2015), whereas the age of the separation of Mesoamerican firs (Sections *Grandis* and *Oiamel*) from those in western North America (Section *Nobilis*) corresponds to the Miocene (Semerikova & Semerikov, 2014). Pollen records show that by the early Pliocene (5.2 million years BP), *Abies* occurred in the area that currently corresponds to the state of Veracruz, in southeast Mexico (Graham, 1999). The fragments of fir forest remaining in western Mexico are relicts of the Pliocene Forest, exhibiting an isolated distribution and replacing the montane cloud forests (MCF) in upper elevations at the western windward of the Trans-Mexican Volcanic Belt (TMVB) (Guerrero-Hernández et al., 2019b). This condition represents an ideal ecosystem to explore the interrelations among climate, microenvironment and structural changes in fir populations and co-occurring species. The canopies of these forests are dominated mainly by *Abies jaliscana* (Martínez) Mantilla, Shalisko & J.A. Vázquez, a relict endemic species that forms nearly monospecific forests at 2300–2420 m a.s.l. (Guerrero-Hernández et al., 2019a) and mix downslope with other tree relict elements of different phytogeographic affinities and typical of the MCF (Vázquez-García, Vargas-Rodríguez & Aragón, 2000; Cuevas-Guzmán et al., 2011; Guerrero-Hernández et al., 2019a). In the western region of Jalisco, Mexico, the illegal logging has become a serious threat to these relict forests, especially in the localities of El Cuale, Cumbre de Guadalupe and El Rosario (*Guerrero-Hernández, personal observation*). Other authors report that illegal logging activities are also reducing the populations of *Abies Jaliscana* (Jalisco fir) at Altamina, Sierra del Cuale (Vázquez-García et al., 20l4).

The climatic and ecological requirements of *Abies* forests in boreal and temperate latitudes are relatively well known (Whittaker, 1960; Cogbill & White, 1991; Taylor & Halpern, 1991; Kneeshaw & Bergeron, 1998; Dobrowolska & Veblen, 2008), however, studies are usually not focused on disentangling the contribution of these environmental factors to the presence of these forests. In subtropical regions, information about climatic and ecological requirements of firs and many threatened tree species along altitudinal zones is better documented (Dang et al., 2010; Linares, Carreira & Ochoa, 2011; Haq et al., 2017; Park et al., 2018); however, accurate data on the size and distribution of Mesoamerican fir species are lacking. Likewise, the relationship of the fir forest structure with microenvironmental and climatic factors has been little studied. For instance, there are studies on *A. hickelii*, *A. jaliscana* and *A. religiosa* in the TMVB (Ávila, Aguirre & García, 1994; Velázquez, 1994; García-Aguirre et al., 2007; Toledo-Garibaldi & Williams-Linera, 2014; Guerrero-Hernández et al., 2019a), however, most of these were conducted in one single or two fir populations and in some cases, firs were not the main subject of the research. There is only one research for Mesoamerican firs, in which several populations of *A. vejarii* and their relationship with environmental and topographic factors were studied in northeastern Mexico (González-Cubas et al., 2020).

Examining the variation in climatic and microenvironmental conditions can help to explain spatial changes in woody species composition, vegetation structure and differences among Jalisco fir populations. Additionally, this knowledge represents an important tool to inform successful conservation and management strategies, contributing to the restoration of endangered forest ecosystems (Linares, Carreira & Ochoa, 2011). Therefore, it is vital to identify structural patterns among western Mexican fir populations and co-ocurring woody species in relation to microenvironmental and climate conditions.

In this study, we explored the stand characteristics, current distribution, and the relationship of climate and microenvironmental factors with vegetation structure of every known forest of *A*. *jaliscana* over their whole distribution range. The specific aims were to: (1) characterize physiognomic structure and size class composition of each western Jalisco fir population, (2) assess the contribution of climate and microenvironmental (abiotic and biotic) conditions that could explain the current structural characteristics of *A*. *jaliscana* populations and co-ocurring woody species, (3) characterize woody species composition in these fir forests, and (4) assess the threat of extinction of this fir species using the IUCN Red List Criteria and propose conservation measures for relict fir populations in western Jalisco, Mexico.

## Materials & methods

### Study area

**Figure 1:**
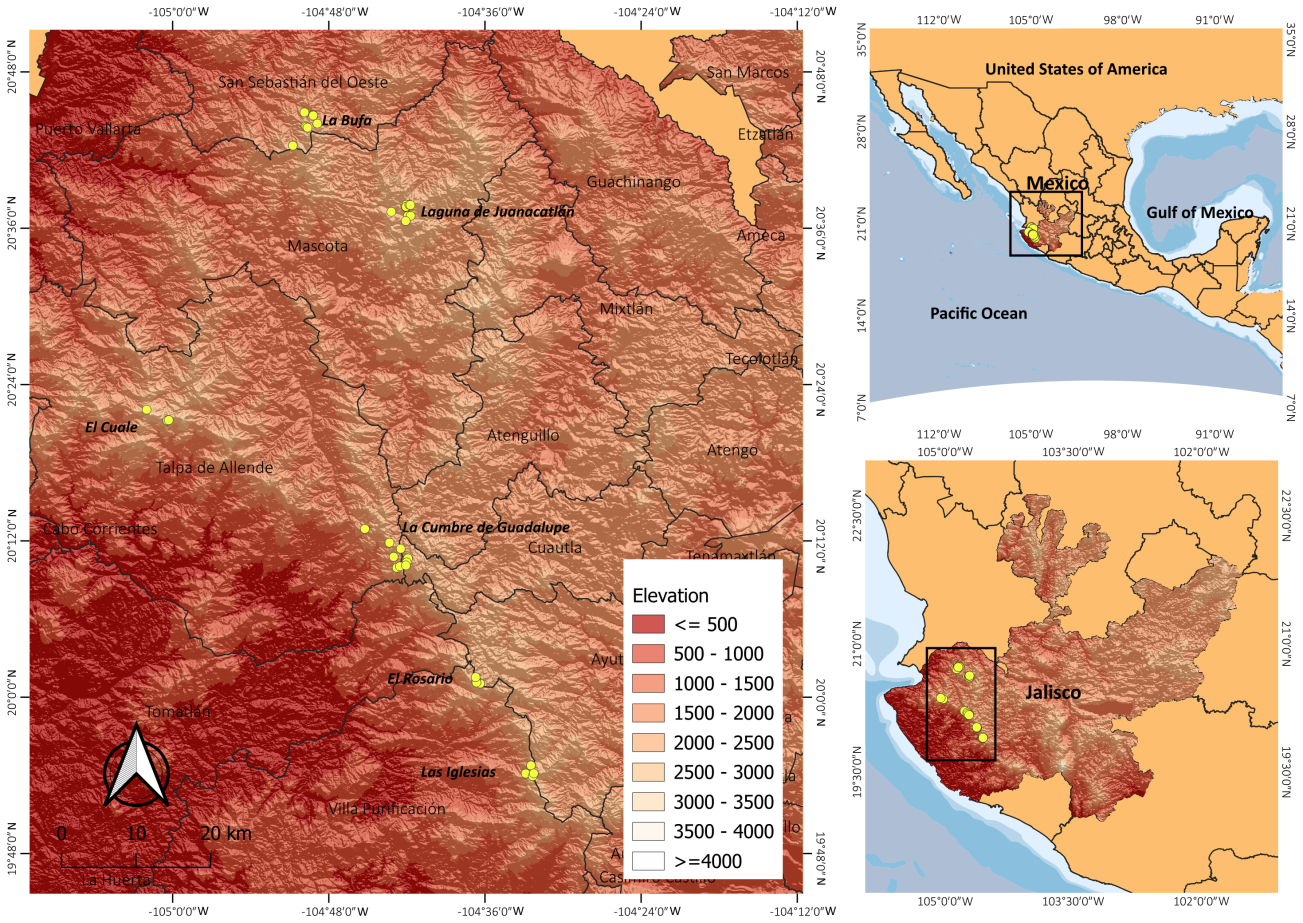
Study area in Jalisco state, western Mexico. White dots indicate study sites.

The study was conducted in the transition zone between the TMVB and Sierra Madre del Sur (SMS) mountain range, in western Jalisco, Mexico. This geographic area is a fraction of the terrestrial ecoregion named “temperate mountains” (CONABIO, 2008). We carried out several field surveys on windward slopes of six localities with fir forest communities in western Mexico (Fig. 1) (map created using a layer from INEGI) (INEGI, 2025): Cerro La Bufa (B) and Laguna Juanacatlán (J), both in the TMVB; Sierra del Cuale (C), Cumbre de Guadalupe (G), El Rosario (R) and Las Iglesias (I), in the SMS. These localities are included in the Jalisco Block, which is the northernmost tectonic block of Sierra Madre del Sur (Ferrari et al., 2000). Rhyolitic tuff from upper Cretaceous-Paleocene and basalt from Pleistocene are the predominant bedrocks to the north, in La Bufa and Laguna Juanacatlán, while in El Cuale, Cumbre de Guadalupe and El Rosario, only rhyolitic tuff from upper Cretaceous-Paleocene is the predominant lithological material. Andesite from the Cretaceous (Albian-Cenomanian) is predominant mainly to the south, in Las Iglesias (Servicio Geológico Mexicano (SGM), 2019).

The nearest meteorological station is located in the town of Cumbre de Guadalupe at 2,120 m of elevation (Servicio Meteorológico Nacional (SMN), 2018), which registered an annual mean temperature of 14.2 °C and a total annual precipitation of 2,003 mm (period 1976–1988). Fir forests in this region are surrounded by oak-pine forests and cloud forest downslopes. Although Western Mexico is a tropical-subtropical transition geographic region (N 20° latitude), due to the elevation, the climate in fir forests is subhumid temperate with summer rains (C (w_2_) (w)) (the wettest of the subhumid and the coldest of temperate climates) (García, 2004). Following the Holdridge life zones system (Holdridge, 1947), these forests correspond to the warm-temperate moist forest zone, but the study sites at the highest elevation are very close to the cool temperate zone and are cooler than other forests that thrive in these highlands. Considering the global bioclimatic classification by Rivas-Martínez, Rivas-Sáenz & Penas (2011), the area is located in the tropical pluviseasonal macrobioclimate, and the Mesotropical and Supratropical belts, with humid ombrotype. The locality Cumbre de Guadalupe exhibits characteristics of a warm-temperate wet forest zone. Based on the WorldClim database, total annual precipitation ranges from 1,330 to 1,725 mm and the mean annual temperature from 13.3 °C, in the upper sites, to 16.3 °C in the lower sites (period 1970–2000) (Fick & Hijmans, 2017). Snowfall is scarce or infrequent; however between 8–10 March 2016, a cold-core vortex spread throughout western and northwestern Mexico, causing snowfall in all localities where *Abies jaliscana* thrives (Servicio Meteorológico Nacional (SMN), 2016).

### Field sampling

We sampled 38 study sites (plots) across a latitudinal geographic range of ca. 100 km and an elevation range of 1,720–2,450 m a.s.l. Sites were distributed among three mountain chains; four sites in the west, within the Sierra del Cuale (C1–C4) 17 in the north region (Sierra de Jolapa) (B1–B8, J1–J9) and 17 in the southern region within the Sierra de Cacoma (G1–G9, I1–I4, R1–R4) (Fig. 1; Table 1).

**Table 1:** Characteristics of the study sites with *A. jaliscana* populations.

**Locality** **code**	**N latitude**	**W longitude**	**Elev (m a.s.l.)**	**Slope (^∘^)**	**AMT (°C)**	**ETRA (mm)**	**AP (mm)**	**IHa**	**Ic**
**La Bufa**									
B1	20°44′53.6″	104°49′51.9″	1,720	34	15.7	775.12	1,511	1.95	5.7
B2	20°44′41.3″	104°49′12.3″	1,970	22	15.3	759.16	1,523	2.01	5.7
B3	20°44′39.2″	104°49′11.6″	2,020	34	15.3	759.16	1,523	2.01	5.7
B4	20°42′20.3″	104°50′45.6″	2,243	18	14.2	717.96	1,559	2.17	5.3
B5	20°43′50.3″	104°49′47.6″	2,306	35	13.7	697.06	1,539	2.21	5.3
B6	20°44′2.2″	104°48′52.4″	2,296	20	14.4	724.62	1,526	2.11	5.7
B7	20°43′47.5″	104°49′43.1″	2,367	31	13.7	697.06	1,539	2.21	5.3
B8	20°43′43.6″	104°49′37.7″	2,447	30	13.7	697.06	1,539	2.21	5.3
**Juanacatlán**									
J1	20°37′15.5″	104°43′11.3″	2,100	28	14.2	711.22	1,480	2.08	5.9
J2	20°36′58.1	104°41′50.4″	2,280	25	13.5	684.48	1,472	2.15	5.9
J3	20°36′57.2″	104°41′42.1″	2,300	18	13.5	684.48	1,472	2.15	5.9
J4	20°36′33.2	104°42′5″	2,342	20	13.8	695.45	1,472	2.12	5.9
J5	20°37′48.4″	104°42′2.2″	2,360	35	13.5	684.50	1,474	2.15	5.8
J6	20°37′36.6	104°42′3.7″	2,374	9	13.5	684.50	1,474	2.15	5.8
J7	20°37′39.2″	104°42′3.2″	2,390	9	13.5	684.50	1,474	2.15	5.8
J8	20°37′48.1″	104°41′48.4″	2,401	12	13.3	677.16	1,473	2.18	5.8
J9	20°37′46.9″	104°41′43.1″	2,413	12	13.3	677.16	1,473	2.18	5.8
**Cumbre de Guadalupe**									
G1	20°12′54.8″	104°45′13.2″	1,794	24	16.3	795.09	1,467	1.85	5.2
G2	20°11′51.4″	104°43′19.8″	2,140	21	14.5	722.09	1,497	2.07	4.8
G3	20°9′56″	104°42′46.3″	2,160	15	15.0	745.80	1,496	2.01	4.8
G4	20°11′23.50”	104°42′28.10”	2,162	22	14.4	716.39	1,478	2.06	5.2
G5	20°10′02.1″	104°42′31.7″	2,172	20	14.8	737.22	1,491	2.02	4.8
G6	20°10′46.1″	104°43′00.6″	2,196	15	15.0	744.62	1,496	2.01	4.8
G7	20°10′38.8″	104°41′54.8″	2,200	10	14.2	710.59	1,477	2.08	5.1
G8	20°10′20.3″	104°41′58.8″	2,260	10	14.4	718.85	1,491	2.07	4.9
G9	20°10′6.7″	104°42′4.20″	2,280	15	14.4	718.85	1,491	2.07	4.9
**El Cuale**									
C1	20°22′4.10″	105°1′59.80″	2,337	33	13.8	714.38	1,725	2.41	5.1
C2	20°21′15.6″	105°0′18”O	2,434	30	13.8	710.83	1,697	2.39	4.6
C3	20°21′12.60″	105°0′23.90″	2,435	30	13.8	710.83	1,697	2.39	4.6
C4	20°21′14.00″	105°0′24.60″	2,445	20	13.8	710.83	1,697	2.39	4.6
**El Rosario**									
R1	20°01′31.5″	104°36′42.5″	2,272	14	14.3	707.75	1,417	2	5
R2	20°01′7.2″	104°36′38.5″	2,334	15	14.2	703.25	1,425	2.03	4.9
R3	20°01′3.1″	104°36′23″	2,338	17	14.1	699.49	1,415	2.02	5.1
R4	20°01′18″	104°36′39.7″	2,343	20	14.2	703.25	1,425	2.03	4.9
**Las Iglesias**									
I1	19°54′7.6″	104°32′53.3″	1,959	37	15.3	732.49	1,330	1.82	5.7
I2	19°54′45″	104°32′27.4″	2,097	33	14.7	713.20	1,342	1.88	5.7
I3	19°53′55.5″	104°32′19.2″	2,167	26	14.4	702.21	1,346	1.92	5.5
I4	19°54′8.4″	104°32′14.5″	2,180	25	14.5	706.36	1,352	1.91	5.6

**Notes.**

Elev Elevation AMT Annual Mean Temperature ETRA Evapotranspiration AP Annual Precipitation IHa Annual Humidity Index Ic Continentality Index

In each study site, we established a stratified random sampling (an area of 60 × 48 m, subdivided in a grid of 12 × 12 m squares) and randomly selected 10 circular subplots or pseudoreplicas (each of 0.01 ha and centered at each square; total area 0.1 ha) (Vázquez & Givnish, 1998). Within these circular subplots, woody individuals alive, dead and stumps ≥ 2.5 cm in DBH (ca. 1.3 m) were tallied, measured and identified at species level. Growth-form (tree, shrub or vine) was recorded for each species. We also recorded latitude, longitude, elevation, aspect and slope, using a GPS (Garmin) and Clinometer (Suunto). A total of 16 environmental variables were recorded at each site, in four subsets: physiographical, disturbance, biotic and climatic variables. No official documents were required from the government agencies that manage natural resources for the fieldwork. Access to the *Abies* forest plots was granted through a verbal agreement with the common land (ejido) members and landowners.

### Data analysis

We calculated density, basal area, relative basal area and relative stem density for each species within the plots (Curtis & McIntosh, 1951). We estimated richness and the Shannon Index using the software Past 4 (Hammer, Harper & Ryan, 2001). We fitted correlations and linear regressions of structural attributes (basal area and density) *vs.* elevation and slope. Based on the size of fir trees, the distribution of Dbh (alive, stump and dead) of each stand was grouped in 10-cm intervals to construct size categories for diameter. For each site, climate information included 19 variables (period 1970–2000) extracted from the WorldClim database (Fick & Hijmans, 2017), which offered a resolution of 1 km (original source: https://www.worldclim.org/data/worldclim21.html#). This resolution is suitable for the scale of our study (the scale is one transect along 100 km from north to south). These variables were screened for collinearity using Pearson correlation coefficients and regression analysis with SIGMAPLOT (Systat Software Inc., 2008). Highly correlated bioclimatic variables were excluded from the ordination analysis; therefore, only six bioclimatic variables were selected. Other climatic variables such as evapotranspiration, annual humidity index (Iha) and simple continentality index (Ic) (Soto, Gama & Gómez, 2001; Rivas-Martínez, Rivas-Sáenz & Penas, 2011) were calculated from the downloaded information. Downloaded climate data were validated in the field with data obtained from the nearest meteorological stations to the study area.

To explore relationships among climatic variables, microenvironmental factors and vegetation structure (fir trees and co-occurring species), we used non-metric multidimensional scaling (NMS), an indirect ordination method useful to evaluate and reduce the dimensionality of a data set (McCune & Grace, 2002). We related the NMS axes to environmental variables and used the Sorensen’s (Bray–Curtis) distance. The variables with scores on either axis larger than a 0.300 cutoff value were plotted to overlay only the most relevant vectors. The main matrix based on basal area of woody species, included 38 study sites and 98 species; the matrix for environmental variables included five physiographical, one of disturbance, one biotic and nine climatic variables. The classification of the study sites was performed with a Hierarchical Cluster Analysis, using woody species basal area and Sorensen (Bray–Curtis) distance measurements, with the beta flexible linkage (beta = −0.25). Ordination and classification analyses were performed using PC-ORD 6.0 (McCune & Grace, 2002). To assess the threat of extinction of the studied fir species based on the International Union for Conservation of Nature-Red List criteria (IUCN-Red List Criteria), we used the GeoCAT application (GeoCAT, 2020) loading the coordinates of our study sites and exporting the data to Google Earth Pro^®^ 7.3.3.7699 (Google, 2020) to generate the layer with the extent of occurrence (EOO) and area of occupancy (AOO). EOO is a parameter that measures the spatial scatter of the areas currently occupied by the taxon, and AOO is a scaled metric that represents the area of suitable habitat currently occupied by the taxon (IUCN, 2001).

## Results

### Forests composition, species richness and classification

A total of 98 woody species representing 59 genera and 40 families were recorded in the 38 study sites. The most species-rich families were Fagaceae (12), Pinaceae (9) and Asteraceae (8). The genera *Quercus* and *Pinus* had several species represented (12 and 8, respectively), while most genera had less than three species. *Abies* and other Holarctic-temperate genera such as *Ostrya*, *Pinus* and *Quercus* were present in more than 95% of the sites. Holarctic, Pantropical and Malayo-American genera (such as *Arbutus*, *Styrax*, *Symplocos* and *Ternstroemia*), occurred in almost 90% of the sites (Appendix S1). Species richness varied from 25 species in La Bufa (B1) to three species in Las Iglesias (I2) and El Rosario (R3). Similarly, Shannon’s diversity index varied from 2.66 in B1 to 0.65 in I2 (Table 2).

**Table 2:** Structural attributes of the studied forests dominated by *A. jaliscana* in western Mexico.

**Study site**	**Fir BA (m^2^/ha)**	**Fir D (stems/ha)**	**Stumps (all tree species)**	**Dead trees (all tree species)**	**Taxa_S**	**Shannon_H**	**Total BA (m^2^/ha)**	**Total D (stems/ha)**
**La Bufa**								
B1	14.09	30	1	0	25	2.66	77.46	1,420
B2	38.93	90	0	3	18	2.55	47.92	820
B3	18.17	80	1	3	17	2.22	41.55	950
B4	42.36	1150	2	11	15	1.99	65.24	2,870
B5	26.19	140	0	4	10	1.72	36.36	540
B6	67.04	530	1	2	13	1.6	67.93	1,040
B7	33.47	280	2	1	9	1.75	55.09	1,060
B8	31.86	130	1	1	13	2.1	66.14	750
**Juanacatlán**								
J1	38.69	350	0	1	18	2.18	74.16	1,800
J2	39.66	500	2	0	17	1.79	60.76	2,430
J3	58.68	590	0	1	12	1.79	68.57	1,480
J4	72.11	140	1	1	13	1.58	79.92	1,230
J5	23.11	160	1	3	15	2.18	54.37	1,450
J6	76.54	530	8	0	9	1.2	80.19	810
J7	76.85	470	4	2	5	0.91	86.94	680
J8	41.67	470	1	1	10	1.46	64.33	1,060
J9	67.49	520	9	2	10	1.59	81.53	1,130
**Cumbre de Guadalupe**								
G1	17.8	70	0	1	22	2.16	42.96	2,160
G2	43.54	280	1	7	13	1.61	63.94	1,340
G3	38.68	290	7	4	4	0.71	47.48	380
G4	52.13	270	6	5	10	1.16	70.46	1,430
G5	78.59	630	1	5	9	1.45	82.69	1,270
G6	46.19	260	2	5	7	1.44	67.23	630
G7	16.4	270	9	16	11	1.84	32.71	670
G8	88.69	350	12	12	5	0.95	96.1	540
G9	64.59	460	0	10	8	1.24	83.36	710
**El Cuale**								
C1	9.17	120	1	10	9	1.73	40.9	1,140
C2	25.5	100	0	7	13	2.02	59.89	910
C3	21.38	160	6	1	9	1.76	44.73	540
C4	30.53	140	0	3	10	1.94	52.48	630
**El Rosario**								
R1	84.65	230	1	2	7	1.37	130.4	400
R2	66.58	180	3	7	8	1.4	89.15	580
R3	56.35	260	5	0	3	0.72	62.44	930
R4	49.76	210	4	3	6	1.16	58.19	340
**Las Iglesias**								
I1	12.29	5	0	0	18	2.2	59.07	1,210
I2	28.67	40	1	3	3	0.65	55.89	550
I3	37.48	8	1	0	9	1.91	76.31	300
I4	31.12	15	5	4	10	1.93	54.62	620

**Notes.**

Fir BA Fir Basal Area Fir D Fir Density Taxa_S Species Richness Shannon_H Shannon Diversity Index Total BA Total Basal Area Total D Total Density

**Figure 2:**
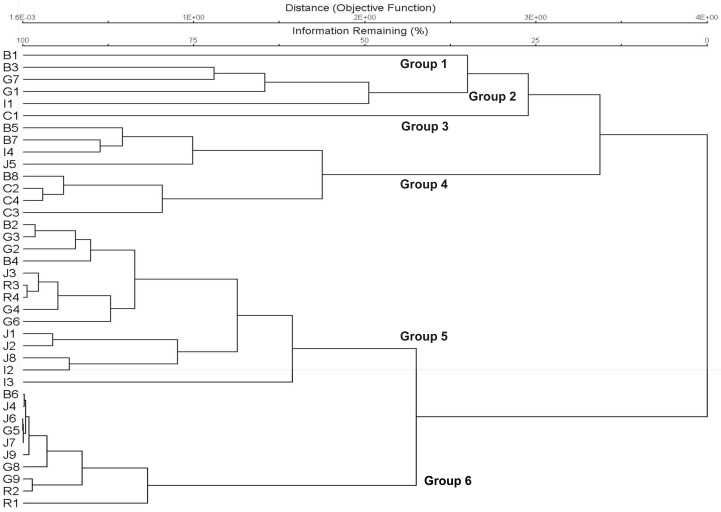
Cluster analysis dendrogram of the 38 study sites using Sorenson distance and beta flexible (−0.25) linkage method, cut at 42.5% of the remaining information scale.

Six plant communities dominated or codominated by *Abies* were identified in the cluster analysis at 42.5% information remaining with a percent chaining = 6.13 (Fig. 2). Each community group was named based on the highest basal area of woody species.

(1) *Magnolia pacifica*-*Abies jaliscana* group: This group consisted of one study site (B1) and 25 species at 1,720 m a.s.l. with a slope of 34°. This study site included elements of both, lower montane cloud forest and the upper montane cloud forest (*sensu* Guerrero-Hernández et al., 2019a).

(2) *Abies jaliscana*-*Quercus centenaria*-*Acer binzayedii* group: This assembly clustered four plots (B3, G1, G7 and I1) with a species richness varying between 22 and 11 taxa distributed between 1,794 and 2,200 m a.s.l., with slopes of 10–37°. This group also included elements of both, lower montane cloud forest and the upper montane cloud forest.

(3) *Pinus strobiformis*-*Pinus pseudostrobus*-*Abies jaliscana* group: This group included only one study plot (C1) and nine species at 2,337 m a.s.l., with a slope of 33°.

(4) *Abies jaliscana-Quercus scytophylla*-*Ostrya virginiana* group: This group comprised eight study plots (B5, B7, B8, C2, C3, C4, I4 and J5) with species richness from 15 to nine woody taxa distributed between 2,180 and 2,445 m a.s.l. with slopes of 25–35°.

(5) *Abies jaliscana*-*Quercus obtusata*-*Cornus disciflora* group: This assembly also included in the understorey several typical elements from the upper montane cloud forest such as *Clethra hartwegii*, *Cleyera integrifolia*, *Meliosma dentata*, *Quercus martinezii*, *Symplocos citrea* and *Ternstroemia lineata*. This community consisted of 14 study sites, with a species richness from 18 (B2 and J1) to 3 woody taxa (R3 and I2), distributed between 1,970 and 2,401 m a.s.l. with slopes of 12–33°.

(6) *Abies jaliscana*-*Pinus montezumae*-*Pinus pseudostrobus* group: In this group, we found the highest basal area and nearly monospecific forests dominated by *Abies*, containing 10 plots, with a species richness varying between 13 and five taxa distributed between 2,172 and 2,413 m a.s.l., slopes of 9–20°.

### Vegetation structure and fir size-class distributions

The woody species with the highest basal area at all localities was *Abies jaliscana*, followed by *Magnolia pacifica* and *Quercus centenaria* at La Bufa; *Q*. *obtusata* for Laguna Juanacatlan, *P*. *pseudostrobus* for Cumbre de Guadalupe, *Q*. *scytophylla* for El Cuale, *Q*. *calophylla* for El Rosario and *Cornus disciflora* for Las Iglesias. Similarly, *A*. *jaliscana* showed the highest abundance (Table S1). We also found high densification of typical elements from cloud forest such as *Styrax argenteus*, *Ternstroemia lineata*, *Symplocos citrea*, *Clethra hartwegii*, *Styrax radians* and *Carpinus caroliniana*, which thrive in the understorey of several study sites at all localities. The individuals of *Abies* with the largest diameters were observed at Laguna Juanacatlan (dbh ≥ 1.30 m).

**Figure 3:**
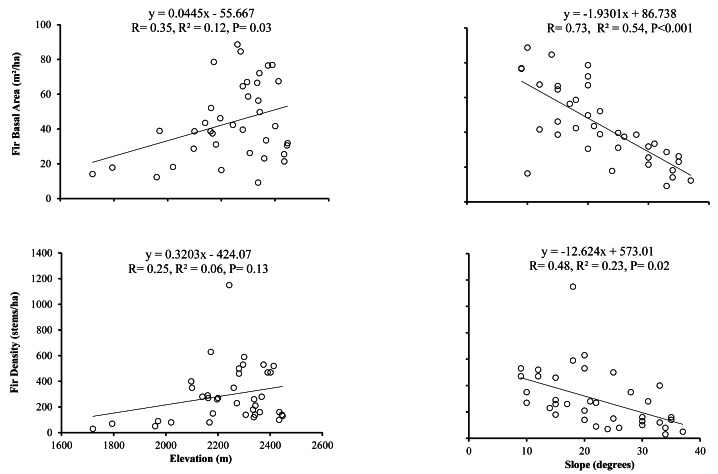
Lineal regression models fitted to structural variables along the elevation range and slope in fir forests of Jalisco, western Mexico.

In terms of basal area per study site, *Abies* accounts for 95.44 and 90.22% of all woody species in sites J6 and J4 respectively (Laguna Juanacatlan), 95 and 92.29% at G5 and G7 (Cumbre de Guadalupe) and 90.24% at El Rosario (R3). Similarly, the highest values in density were recorded for *Abies*, which accounts for 76% at Cumbre de Guadalupe (G3), 72% at Las Iglesias (I2) and 69.11% at Laguna Juanacatlan (J7). However, study sites G3 and I2 showed a very low fir basal area, whereas J7 exhibited a high percentage of fir basal area; both structural variables are shown in absolute values in Table 2. Densities of fir trees in relation to elevation did not show a significant trend (ANOVA, *F* = 2.398, *P* = 0.130), while in relation to slope, they displayed statistically significant differences (ANOVA, *F* = 10.713, *P* = 0.02) and a monotonic linear decrease. On the other hand, fir basal area exhibited a significant increase in relation to elevation (ANOVA, *F* = 4.928, *P* = 0.033) and slope (ANOVA, *F* = 41.573, *P* < 0.001) (Fig. 3).

**Figure 4:**
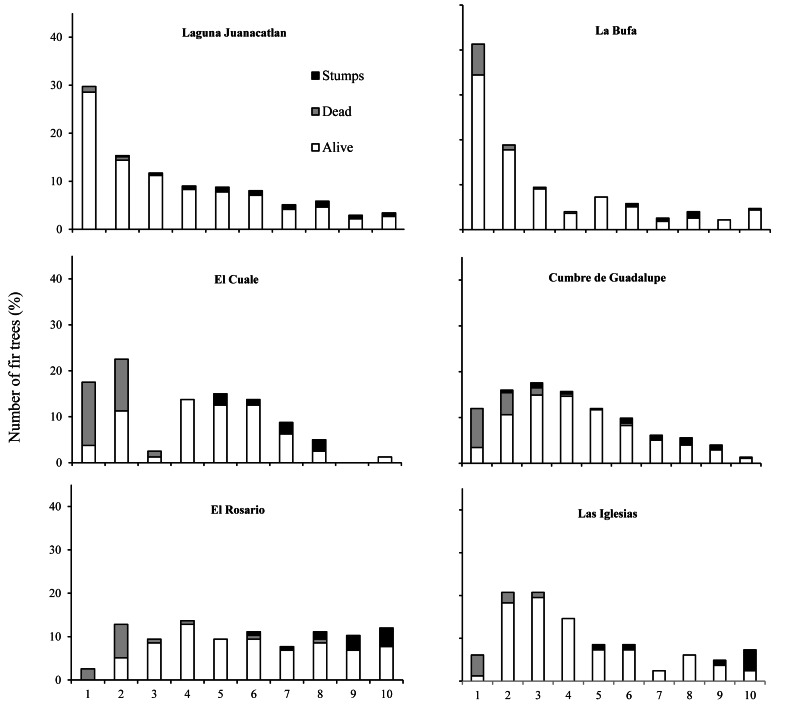
Diameter class distribution (dbh, cm) for western Mexican fir trees at Laguna Juanacatlán, La Bufa, El Cuale, Cumbre de Guadalupe, El Rosario and Las Iglesias. Each number indicates a diameter interval, (1) 2.5–9.9 cm, (2)10–19.9 cm. (3) 20–29.9 cm. (4) 30–39.9 cm. (5) 40–49.9 cm. (6) 50–59.9 cm. (7) 60–69.9 cm. (8) 70–79.9 cm. (9) 80–89.9 cm (10) ≥ 90 cm.

Size-class distributions of fir trees differed among localities. An inverted-J pattern of diameter distribution of firs was observed at two localities; La Bufa and Laguna Juanacatlán. At these sites, a high percentage of alive and dead fir trees occurred in the smallest diameter categories (Fig. 4). In contrast, at Laguna Juanacatlán, stumps occurred in all diameter categories, whereas in La Bufa, stumps occurred in one of the largest categories. Fir diameter distribution showed a bell-shaped pattern at Cumbre de Guadalupe, El Cuale and Las Iglesias, with a peak in the categories 2, 3 and 4 (dbh 10–39.9 cm), whereas an irregular pattern was observed for El Rosario, with a higher percentage of alive trees in the two largest categories compared to the rest of localities (Fig. 4). In these last four localities, the highest percentage of stumps occurred in the largest diameter categories, except at Cumbre de Guadalupe. In this locality, there were stumps in all diameter categories, with a peak in the category 8. In contrast, fir dead trees occurred in the smallest diameter categories.

### Relationship between vegetation structure and microenvironment- climate

**Figure 5:**
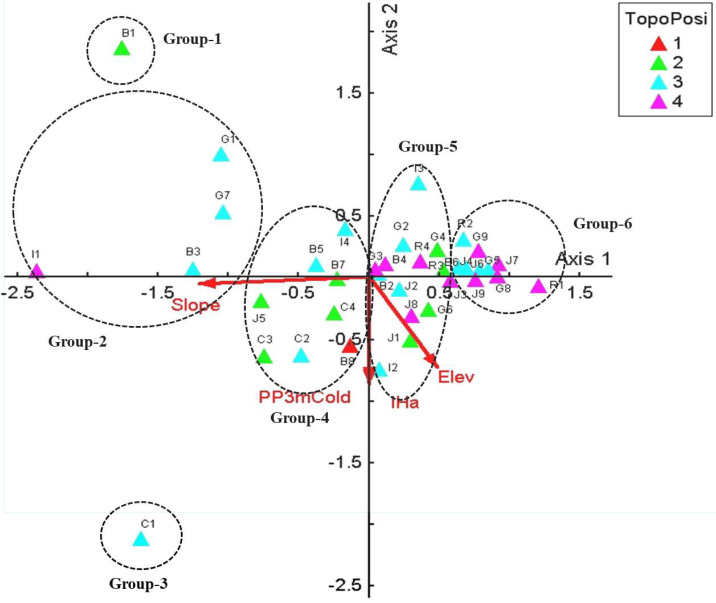
Non-metric Multidimensional Scaling analysis (NMDS), using the 38 study sites (final stress 12.66) at each locality in western Jalisco, Mexico. Numbers and triangles are forest sites. Arrows (vectors) are significant variables (scores on axes larger than the 0.300 cutoff value): slope, annual humidity Index (IHa), elevation (Elev), topographic position (TopoPosi), and precipitation of coldest quarter (PP3mCold).

From 38 study sites and based on basal area of woody species, results of the NMS analysis showed a final stress of 12.66 with 250 iterations, for a two-dimensional solution. Most of the stress was reduced after 62 iterations. Elevation, annual humidity index, precipitation of coldest quarter of the year and slope, were the variables with scores on axes larger than the 0.300 cutoff values (Fig. 5). Variance in the first axis was mainly correlated with two physiographic variables and three climatic variables. Variance in axis 2 was explained by one physiographic and five climatic variables (Table 3). Nearly monospecific fir forests were located in the right extreme position of Axis 1. Some study sites with smaller fir basal area and with a dense understorey, composed of cloud forest elements, were distributed in the left extreme position and along axis 2 (B1 and C1) (Fig. 5).

**Table 3:** Climatic and microenvironmental variables with Pearson correlation coefficients (r) in each ordination axe, using basal area data of woody species dbh ≥ 2.5 cm from the 38 study sites with fir forest.

	Variable	Basal area data	
Physiographic		Axis 1 (r)	Axis 2 (r)
	Elevation	0.442	−0.546
	Slope	−0.7	−0.158
	Rad Pot	0.28	0.103
	Heat load	0.179	0.299
	Stoniness	−0.453	0.131
Disturbance			
	Stumps	0.234	0.086
			
Biotic			
	Dead trees	−0.015	−0.138
			
Climatic			
	Annual Mean Temperature	−0.312	0.46
	Annual Precipitation	−0.247	−0.129
	Precipitation of Driest Quarter	0.207	−0.261
	Precipitation of Coldest Quarter	−0.014	−0.589
	Evapotranspiration	−0.305	0.416
	Annual Humidity Index	0.017	−0.594
	Temperature Annual Range	−0.074	0.461
	Precipitation of Wettest Quarter	−0.434	−0.032
	Continentality Index	0.17	−0.019

**Notes.**

Rad Pot, Potential annual direct incident radiation.

The strongest correlation occurred between these two study sites and slope; the decline in fir basal area was associated with increases in slope and decreases in elevation (Table 2; Figs. 3 and 5). In contrast, high values of fir basal area were inversely correlated with slope, and some of these study sites are also located at high elevations (*e.g.*, J6, J7, J9 and R2) (Table 1). The highest correlations along axis 2 were the annual humidity index and elevation (Table 3); high values of these variables were associated to study sites with *Quercus scytophylla*, *Pinus herrerae* and *P. strobiformis*. These variables also explain the largest basal area of *Arbutus xalapensis*, *Quercus laurina*, *Q. obtusata* and *P. pseudostrobus* (Table 4 and Table S1), achieving a balance in dominance with *Abies*.

**Table 4:** Dominant tree species with Pearson correlation coefficients (r) in each ordination axe, using basal area data ≥ 2.5 cm from the 38 study sites.

Woody species	Axis 1 (r)	Axis 2 (r)	Woody species	Axis 1 (r)	Axis 2 (r)
*Abies jaliscana*	0.891	0.084	*Pinus pseudostrobus*	−0.062	−0.468
*Acer binzayedii*	−0.212	0.285	*Pinus strobiformis*	−0.327	−0.607
*Arbutus xalapensis*	0.052	−0.401	*Quercus calophylla*	0.102	0.084
*Carpinus caroliniana*	−0.406	0.592	*Quercus castanea*	0.093	−0.126
*Clethra hartwegii*	−0.5	0.005	*Quercus centenaria*	−0.384	0.044
*Cleyera integrifolia*	0.073	0.121	*Quercus laurina*	−0.111	−0.259
*Cornus disciflora*	0.059	0.253	*Quercus martinezii*	0.062	0.209
*Magnolia pacifica*	−0.355	0.532	*Quercus nixoniana*	0.156	0.062
*Meliosma dentata*	−0.046	0.096	*Quercus obtusata*	0.185	−0.241
*Ostrya virginiana*	−0.334	0.001	*Quercus scytophylla*	−0.203	−0.294
*Pinus douglasiana*	0.033	0.074	*Styrax argenteus*	0.218	−0.162
*Pinus herrerae*	−0.27	−0.584	*Symplocos citrea*	−0.12	0.263
*Pinus montezumae*	0.347	0	*Ternstroemia lineata*	0.155	−0.044

### Conservation status

**Figure 6:**
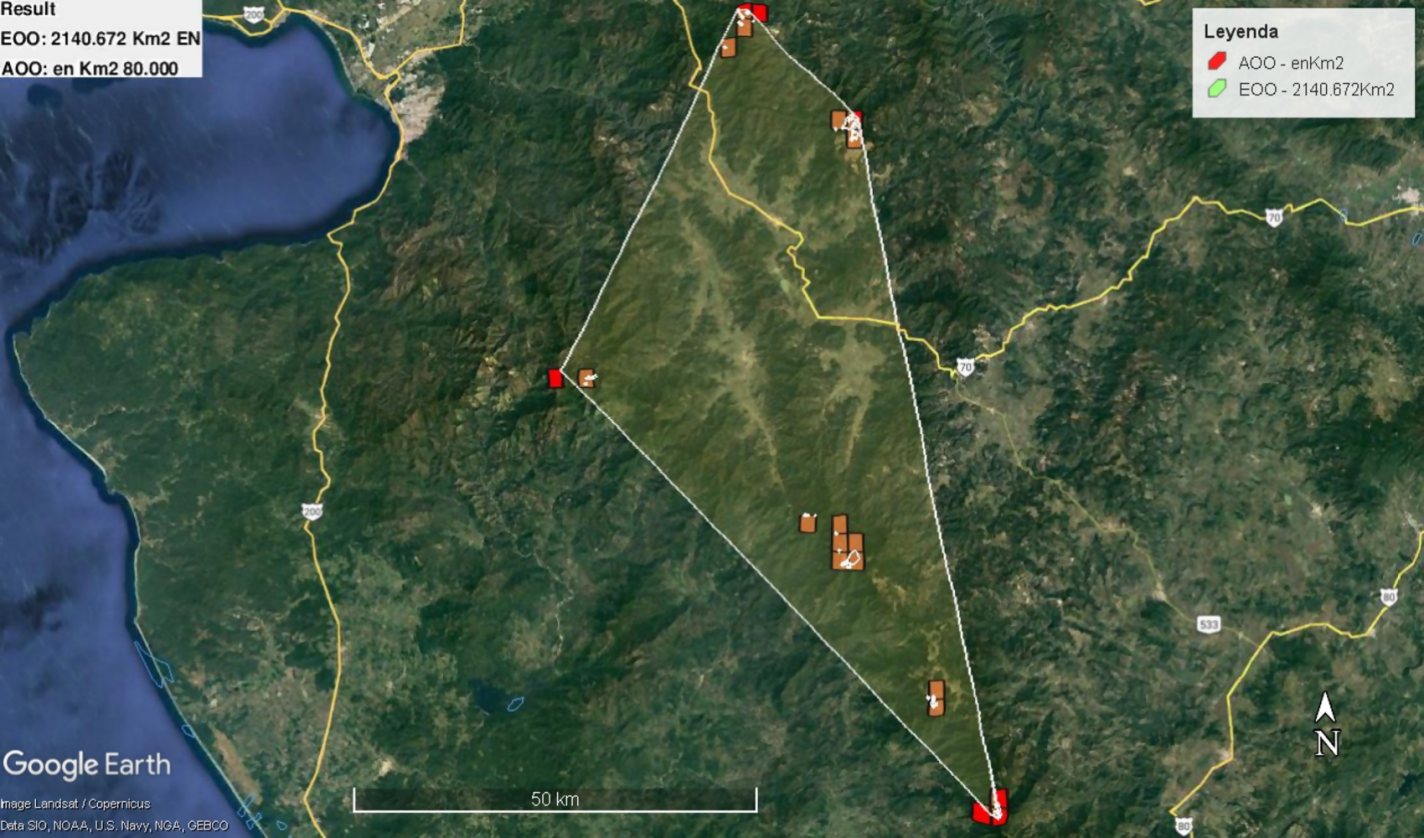
Extent of Occurrence (EOO) and Area of Occupancy (AOO) of *Abies jaliscana* in western Jalisco, México.

The relict forests dominated by *A*. *jaliscana* have an extent of occurrence (EOO) of 2,140.67 km^2^, and area of occupancy (AOO) of 80 km^2^ (Fig. 6). In accordance with these results, the IUCN Red List criteria Bl, B2, and conditions [ab_(ii,iii,v)_], the conservation assessment for this species is under the category of “Endangered” (EN) (IUCN, 2012), given that the EOO is < 5,000 km^2^ (criterion B1) and the estimated AOO < 500 km^2^ (criterion B2) (Fig. 6). Furthermore, it is distributed in only six severely fragmented populations [condition (a)] (Fig. 6), that are undergoing a continuing decline [conditions (b) _(ii,iii,v)_], in area of occupancy _(ii)_, area and quality of habitat _(iii)_, and number of mature individuals _(v)_ (IUCN, 2012). This continuing decline is due to a constant anthropogenic destruction and degradation. Illegal timber extraction, led by organized crime gangs in the last ten years in western Mexico, has been a notorious cause of forest degradation (García-Jiménez & Vargas-Rodríguez, 2021; Torres-Rojo, 2021). In addition to timber extraction in these Jalisco fir forests, there are adjacent large-area plots totally converted from forest to farm land (Guerrero Hernández, personal observation). Natural fires are also a significant danger of great-scale disturbance, which are recurrent in drier slopes of western Mexico. The surface affected by fires in Mexico has duplicated from 849,632 hectares in 1998 to 1′672,216 in 2024 (CONAFOR, 2025). Finally, Other 23 woody species were also found under legal protection (Table 5).

**Table 5:** Status of native and endemic woody species under legal protection based on the criteria of the IUCN and the Mexican Endangered Species Act (NOM-059).

Species	Family	Status		GD
		IUCN	NOM-059	
*Abies jaliscana*	Pinaceae	**NT**	P	Endemic
*Acer binzayedii*	Sapindaceae	**CR**	P	Endemic
*Carpinus caroliniana*	Betulaceae	LC	A	Native
*Clethra hartwegii*	Clethraceae	LC		Endemic
*Cleyera integrifolia*	Pentaphylacaceae	LC		Endemic
*Cornus disciflora*	Cornaceae	**VU**		Native
*Crataegus mexicana*	Rosaceae	LC		Endemic
*Ilex tolucana*	Aquifoliaceae	**VU**		Native
*Juglans major*	Juglandaceae	LC	A	Native
*Magnolia iltisiana*	Magnoliaceae	**VU**	A	Endemic
*Magnolia pacifica*	Magnoliaceae	**EN**		Endemic
*Ostrya virginiana*	Betulaceae	LC	Pr	Native
*Persea hintonii*	Lauraceae	**VU**		Endemic
*Pinus douglasiana*	Pinaceae	LC		Endemic
*Pinus herrerae*	Pinaceae	LC		Endemic
*Pinus strobiformis*	Pinaceae	LC	Pr	Native
*Podocarpus matudae*	Podocarpaceae	**VU**	Pr	Native
*Quercus acutifolia*	Fagaceae	**VU**		Native
*Quercus martinezii*	Fagaceae	LC		Endemic
*Quercus obtusata*	Fagaceae	LC		Endemic
*Quercus scytophylla*	Fagaceae	LC		Endemic
*Senna multifoliolata*	Fabaceae	**VU**		Endemic
*Tilia americana* var. *mexicana*	Malvaceae	LC	P	Native

**Notes.**

Black letters indicate the most relevant IUCN categories. (LC) Least Concern, (NT) Near Threatened, (VU) Vulnerable, (EN) Endangered, (CR) Critically Endangered, (Pr) Special protection, (A) Endangered (NOM-059), (P) Danger of extinction, (GD) Geographical distribution.

## Discussion

### Floristic composition and species richness

We recorded a high diversity—above 15 woody species ≥ 2.5 cm dbh in some study sites—where *Abies jaliscana* is the dominant species (Table 2), as reported in previous studies (Cuevas-Guzmán et al., 2011; Guerrero-Hernández et al., 2019b). We suggest that this pattern occurs due to wide elevation differences in the distribution of Jalisco fir forests. Floristic patterns and ecological processes in *Abies*-dominated forests have been studied worldwide (*e.g.*, Tang & Ohsawa, 1997; Linares, Carreira & Ochoa, 2011; González-Cubas et al., 2018) and a clear trend documented is that *Abies*-dominated forests usually co-ocurr with other conifer and broadleaves temperate and boreal taxa. Differentiation in the composition of woody species varies based on the geographical distribution of these forests. In subtropical latitudes of North America, fir species co-occur with woody taxa of Holarctic, Neotropical and Southeast Asia (Malayo-American or Amphi-Pacific *sensu* Van Der Hammen & Cleef (1983)) affinity, depending on the elevation (Ohsawa, 1995; Olvera-Vargas, Figueroa-Rangel & Vázquez-López, 2010; Guerrero-Hernández et al., 2019b).

In Mexico, fir forests have been considered reservoirs of high similarity and low floristic diversity, especially tree and woody taxa (Rzedowski, 1978). For instance, in northeastern Mexico, González-Cubas et al. (2018) studied four *Abies vejarii* forests, which exhibited tree-species richness of 6–11 and a Shannon diversity index from 1.47 to 1.76. The relatively low diversity in northern Mexico could be explained by the null presence of tropical tree taxa and the high incidence of frost and snowfall from November to April (Giménez de Azcárate, Macías Rodríguez & Gopar-Merino, 2013). On the other hand, Sánchez-González, López-Mata & Granados-Sánchez (2005) reported that distribution of species richness is not uniform among localities with sacred fir forest (*A*. *religiosa*) in the TMVB, and tree richness was low. The trend with low plant richness, is generally reported in studies that do not consider elevation range where the fir species is dominant or co-dominant, *e.g.*, forests of *A*. *religiosa* subsp. *colimensis* with six to nine woody species/plot 0.1 ha (Cuevas-Guzmán et al., 2011). There are other cases with low diversity (0.10–1.34 Shannon diversity index) related to a high densification by the dominant fir, as in *A*. *pinsapo* forests in the Mediterranean basin (Linares, Carreira & Ochoa, 2011), although latitudes above 34°N allow a high incidence of frosts which would be a discriminant factor in plant richness. Therefore, based on our results and previous studies, environmental and elevational gradients need to be considered to study the ecological characterization of any plant community.

### Vegetation structure and fir size-class distributions

At all study sites, the most important species in terms of structure was *A. jaliscana*, except in some plots of low elevation at El Cuale. Therefore, the basal area and density of this taxon were different among localities; basal area was higher at Laguna Juanacatlán, Cumbre de Guadalupe and El Rosario, which can be considered as mature and nearly monospecific forests, except in low-elevation study sites (J1, J2 and G1). The dominance in terms of basal area of *Abies*, varies from being the only tree species to form part of mixed forests, where these fir trees are codominant in the canopy and understorey due to differences in elevation, temperature and slope (Tables 1 and 2; Fig. 5). Some studies in Asia, Europe, eastern and western Mexico have shown similar results, where dominance of *A*. *fabri*, *A*. *fargesii*, *A. jaliscana*, *A*. *pinsapo* and *A*. *religiosa* varies in relation to elevation (Sánchez-Velásquez, Pineda-López & Hernández-Martínez, 1991; Arista, 1995; Tang & Ohsawa, 1997; Dang et al., 2010; Guerrero-Hernández et al., 2019a). In contrast, firs in northern Mexico cover smaller forest stands than those distributed in the TMVB; their basal area is lower than most of the *Abies* in our study area, from 2.6–16 m^2^/ha south of Nuevo León, to an average of 16.44 m^2^/ha in Sierra de Zapalinamé, Coahuila, where *A*. *vejarii* thrives (Encina-Domínguez et al., 2008; González-Cubas et al., 2018). The lower BA in *A*. *vejarii* forests compared to our study area, could be related to high water stress (560–600 mm in Coahuila and 800 mm in Nuevo León) and high competition with other conifers that thrive in both cool-temperate humid and subhumid climates. Likewise, in northwestern Mexico, basal area values recorded for *A*. *durangensis* vary from 0.2–84.1 m^2^/ha, however, almost all plots were below 50 m^2^/ha, except one with a value above 50 m^2^/ha (Quiñones Pérez, Silva-Flores & Wehenkel, 2012). In contrast, in our study, 13 plots (34.2% of the study sites) showed fir BA values above 50 m^2^/ha, even reaching values as high (96 m^2^/ha) as those in forests dominated by *Abies magnifica* (red fir) in northwestern California (Taylor & Halpern, 1991). Precipitation above 1000 mm in some habitats of *A*. *durangensis* is very favorable, but high competition with *Picea chihuahuana*, *Pinus strobiformis*, *P*. *durangensis*, *Pseudotsuga menziesii*, *Quercus scytophylla* and *Q*. *sideroxyla*, promotes significant changes in vegetation structure along its latitudinal distribution, resulting mostly in mixed-coniferous and few monospecific fir forests. In general, we have observed that fir species in northern Mexico are not distributed over wide elevational gradients, whereas fir species in the TMVB are distributed in wide altitudinal intervals with higher precipitation, allowing higher biomass and tree size.

The studied localities showed contrasting patterns in diameter classes of *Abies jaliscana* over their whole distribution range. In Laguna Juanacatlán and La Bufa, the reverse J-shape distribution of diameter classes (Fig. 4) indicates that the forest has favorable regeneration and recruitment potential. Some authors have also found a reverse J-shape size distribution in other Mexican fir forests, suggesting that these forests are multi-aged with several shade-tolerant woody species that are recovering from logging and other disturbances (Encina-Domínguez et al., 2008; Cuevas-Guzmán et al., 2011). In contrast, a bell-shape and an irregular pattern were also found in the rest of the studied localities. The bell-shape pattern in Cumbre de Guadalupe could imply poor regeneration and recruitment due to seedling mortality, recruitment limitation and environmental constraints caused by habitat fragmentation, land-use change and excessive logging. This pattern must be addressed, since species with little saplings or low natural regeneration are usually under threat of local extinction (Mewded, Negash & Awas, 2020). As with other tree-like and woody species, the irregular pattern could be associated with non-selective logging, since any diametric size is used for house construction, timber, farm tools, and firewood (Kala, 2015; Goncalves et al., 2018).

### Relationship between vegetation structure and microenvironment- climate

Our data support the hypothesis that distribution boundaries are driven by environmental conditions associated with elevation and slope. These variables and annual humidity index are influential in plant community structure and woody species composition, whereas variation in aspect (heat load and potential annual direct incident radiation), stoniness, dead trees and anthropogenic disturbance (stumps) do not play a significant role. However, there is evidence of severe logging in the recent past on the plot B4; we found high densification of fir trees, rotten stumps and lower BA compared to some plots with low density such as J7, G8 and R1 (Table 2). The simple continentality index was not significantly correlated with vegetation structure because all localities harbor fir trees, exhibit high precipitation (above 1,000 mm) and a very low continentality (4.6–5.9) (Table 1), therefore, they are classified as Euhyperoceanic (*sensu* Rivas-Martínez, Rivas-Sáenz & Penas, 2011). The condition of low continentality maintains high humidity and low thermal oscillation throughout the year. Values for the continentality index are very similar to those reported for other fir species that occur in the TMVB, such as *A*. *religiosa* in Los Azufres, Michoacán (Ic = 5.5), but are lower than those reported for fir species in Sierra Madre Occidental (Giménez de Azcárate, Macías Rodríguez & Gopar-Merino, 2013). The climatic differences are very slight, however, elevation directly influences other climatic variables such as temperature, humidity, winter rainfall and evapotranspiration. Elevation affects species distribution, structure and diversity among forest communities (Trejo & Dirzo, 2002; Mewded, Negash & Awas, 2020) and in our study area, fir forest subtypes as well. Similarly, other studies have reported elevation as a main factor for woody and tree species distribution in fir forests (*e.g.*, Ávila, Aguirre & García, 1994; Guerrero-Hernández et al., 2019a). It has been found that the high correlation between elevation and dominance-presence of sacred fir (*Abies religiosa*) forest in central Veracruz, Mexico, is due to the broad elevational range of 4,000 m which includes other types of non-*Abies* plant communities at lower elevations, such as cloud forest and tropical dry forests (Toledo-Garibaldi & Williams-Linera, 2014). Similarly, the presence of *A*. *pindrow* in the Himalayan region of Pakistan is positively correlated with elevation (Ahmed, Shahid-Shaukat & Faheem-Siddiqui, 2011). In this study, the relation between elevation and climate is relatively low since each forest patch is located in a different micro basin along 100 km from north to south and the total elevational range is less than 1,000 m. Likewise, latitude is another factor to take into account; for instance, La Bufa, Laguna Juanacatlán (northernmost localities) and El Cuale, exhibit slightly colder temperatures than the southern localities Cumbre de Guadalupe, Las Iglesias, and even El Rosario, which is located in similar elevation belts as those for localities B and J (Table 1).

It has been shown that elevation and slope, play a fundamental role in determining woody and tree species composition at a local and regional scale (Williams-Linera, Toledo-Garibaldi & Gallardo-Hernández, 2013; Guerrero-Hernández et al., 2019a). In this research, slope had the greatest influence on forest communities; therefore, the sites with the lowest fir basal area (I1, B1, B3, J5) and most of all those study sites with Fir BA < 32 m^2^/ha (Table 2; Fig. 5), exhibited the highest slope values. Steep slopes and low elevation appear to affect negatively these fir populations, and are related to higher temperatures and likely shallow soils, restricting the fir basal area due to densification and competition with other Holarctic broadleaved tree taxa such as *Acer*, *Carpinus*, *Magnolia* and *Quercus*. Conversely, gentle slopes could be related to deep soils, allowing high fir basal area due to better anchoring of tree roots; incidentally, these characteristics were found in some higher elevation study sites such as R2, R3, J6, J7 and J9. Hence, high elevation and gentle slope could influence simultaneously on a better growth and development of fir trees. In contrast, other studies have reported that neither slope nor environmental variables have a strong effect on any of the response variables. For instance, Linares, Carreira & Ochoa (2011) found that anthropogenic disturbance explains the highest percentage of the variance in *A. pinsapo* forests in south Spain, despite that elevation and aspect provided a significant increase in the total sum of eigenvalues. On the other hand, a negative correlation between fir density and specific environmental variables has also been reported. For instance, in a study addressing the regional decline of *A. koreana* in the Korean peninsula, Park et al. (2018) found that density was negatively associated with slope aspect, topographic position index, *Quercus mongolica* cover, and mean summer temperature. In our study, the annual humidity index (Fig. 5) and precipitation variables (annual, winter rainfall and summer rain) (Table 1)—which have a lower correlation than slope and elevation—also contribute to explain differences in vegetation structure and presence of other taxa like *Pinus strobiformis* in only one locality, which is more abundant in northern Mexico and south of the USA. Similarly, other studies have found average annual and winter rainfall increase trends in relation to elevation, contributing to the presence and dominance of *Abies jaliscana*, *A*. *religiosa* and *P*. *hartwegii* (Reich et al., 2010; Guerrero-Hernández et al., 2019a; Guerrero-Hernández et al., 2019b). However, it is necessary to emphasize that there is a lack of studies that assess the influence of environmental variables on the presence of conifer communities. Winter precipitation seems to be determinant for temperate-like taxa that thrive at low latitudes and high elevations.

We did not take into account edaphic variables because in a previous research we found the highest correlations among some of these variables and pine-oak and cloud forests (only one study site for the latter) (Guerrero-Hernández et al., 2019a). Furthermore, fir forests or mixed coniferous forest are mainly a type of zonal vegetation (mostly influenced by climatic factors), however, soil could explain the presence of other plant life-forms such as myco-heterotrophs (*e.g.*, *Hexalectris grandiflora*, *Monotropa hypopitys*, *Orthilia secunda*), which are common in these types of well-preserved forest communities (Sánchez-González, López-Mata & Granados-Sánchez, 2005; García-Arévalo, 2008; Guerrero-Hernández, González-Gallegos & Castro-Castro, 2014). In general, Mesoamerican fir species are humidity-demanding, although most of the species avoid habitats with stagnant moisture (Semerikova & Semerikov, 2014). They thrive in temperate and cool climates with low thermal oscillation, with some exceptions such as *Abies durangensis* and *A*. *vejarii*, which thrive in sites with six-month frosts and extreme minimum temperatures (−15 and −14 °C respectively) (Giménez de Azcárate, Macías Rodríguez & Gopar-Merino, 2013; Servicio Meteorológico Nacional (SMN), 2021). All of these features determine the zonal affinity of firs as main elements in the upper zone of the mountain range.

### Conservation

Taxonomic treatments of Mexican fir species have been very confusing and contrasting causing errors and inconsistencies in the risk assessment of Mexican government agencies (applying the NOM-059) and the International Union for Conservation of Nature (IUCN) Red List. *Abies guatemalensis* subsp. *jaliscana* (Martínez) Silba is a synonym of our target species *A. jaliscana* (Tropicos, 2019), which has been designated as “special protection” and “endemic” in the update of the Mexican Endangered Species Act NOM-059 (SEMARNAT, 2010; Diario Oficial de la Federación (DOF), 2019); this policy refers to it as “Jalisco Fir”. Additionally, this norm also considers the valid species *A. flinckii* Rushforth as a synonym of *A. guatemalensis* subsp. *jaliscana*. It is necessary to emphasize that *A. flinckii* and *A. guatemalensis* are different species to *A. jaliscana* (Rushforth, 1989; Vázquez-García et al., 2014). In this research we followed the proposal of Vázquez-García et al. (2014), considering *Abies jaliscana* as a valid endemic species, which is restricted to six fragmented localities in western Jalisco, Mexico. In the current IUCN Red List, *A. jaliscana* appears as “Near Threatened” (Thomas, 2013), but this assessment needs to be updated because it included a population of *A. flinckii* from the state of Michoacán, misestimating the EOO range up to 15,000 km^2^. The estimated EOO in this research is 2,140.67 km^2^, confined only to western Jalisco. Thus, *Abies jaliscana* deserves the IUCN Red List category of “Endangered” because it has an EOO < 5,000 km^2^ and an AOO < 500 km^2^, in addition to have only six severely fragmented populations and a continuous decline in AOO and quality of its habitats. Hence, the current IUCN Red List category for this Jalisco fir species needs to be updated. Besides the limited range and area of occupancy of this species and its severely fragmented populations, the main threat causing its continuous population decline is illegal logging. Over the past 10 years, organized crime gangs have been logging extensive parts of the forests in western Mexico with total impunity and on an ongoing basis (García-Jiménez & Vargas-Rodríguez, 2021; Torres-Rojo, 2021). Therefore, it is necessary to urge authorities at all levels of government to fulfill their responsibility to enforce forest and environmental protection laws. The congeneric species *Abies guatemalensis*, which faces similar threats and AOO to *A. jaliscana* has been assessed at the IUCN Red List as Endangered (Gardner, 2013).

Based on criteria of territorial area covered by *Abies jaliscana* and our results from six identified populations, we propose two protective legal statuses for its conservation. First, we suggest that *A. jaliscana* must be urgently included in the IUCN Red List under the category of “Endangered” (EN) (Bl, B2, ab _(ii,iii,v)_, IUCN Red List Categories and Criteria) (IUCN, 2012). Second, the forest of Laguna Juanacatlán contains the largest and most resilient of Jalisco fir populations, hence it should be included either as a protected area, as a biosphere reserve in the federally protected natural areas system, which are managed by Comisión Nacional de Areas Naturales Protegidas (CONANP), or at least decree the area as a state park. All these forests have unprotected legal status, with the exception of La Bufa and one small fragment in the Laguna Juanacatlán locality which lies within the Río Ameca Natural Resources Protection Area. We urge to stop all kinds of logging (legal and illegal), since the importance of preserving these forests is too high due to their biogeographic history, and the natural and hydrological resources they provide to the environment. Likewise, Mesoamerican firs have different genetics and evolutionary traits, compared to those in higher latitudes in North America and Asia (Semerikova & Semerikov, 2014; Xiang et al., 2015), that need to be preserved.

Given the threats this Jalisco fir species faces, it is necessary to continue studying its population ecology and ecophysiology in order to obtain basic knowledge for its survival, reproduction, and reintroduction to the wild. The specific environmental and climatic requirements of this fir species (high rainfall, cool temperatures, low thermal oscillation, gentle slopes and its restriction to the north and windward slopes in mountainous areas) makes it vulnerable to climatic fluctuations. Therefore, conservation actions that take into account this vulnerability are recommended. The threatening situation of Jalisco fir populations requires the creation of incentives for forest landowners to implement fir breeding *in situ* and *ex situ* production programs. The Jalisco fir is a species in high demand as a timber product, therefore, its current populations must be preserved. We must encourage production of seedlings in forest nurseries for their reintroduction into natural populations, to restore forest cover where deforestation occurred due to agricultural or livestock activities. Finally, similar to other fir species that thrive in temperate and cool forests in tropical-subtropical America, the restricted range of *Abies jaliscana* and its fragmented pattern of distribution partially evidence its relictual trait.

## Conclusions

Our results indicate that the distribution limits of *Abies jaliscana* forests are driven by climatic and microenvironmental conditions associated with elevation and slope. Analysis of the structure of the six Jalisco fir populations, suggest a multiaged population with good regeneration and some populations with signs of disturbance. Despite its restricted distribution, *Abies jaliscana* is locally dominant at the highest elevations in the mountains of western Jalisco with a high regeneration. Western-Mexican fir forests not only harbor a high number of woody species, as we have quantified here, but also most of them are endemic, relict and endangered species (*e.g.*, *Acer binzayedii*, *Magnolia iltisiana* and *M*. *pacifica*); furthermore, some of these taxa are located at their southernmost distribution range (*e.g.*, *Pinus strobiformis*).

Based on our conservation assessment we urgently recommend restoration and conservation practices, which must be applied mainly to Cumbre de Guadalupe, El Rosario and Las Iglesias. The present research provides the first data on variation in climate and microenvironmental conditions along the whole distribution of *Abies jaliscana* forests in western Mexico.

## Supplemental Information

10.7717/peerj.21342/supp-1 Supplemental Information 1 List of families and species of woody plants in 38 study sites of fir forest in western Jalisco, Mexico Values are basal area of woody species in each site.

10.7717/peerj.21342/supp-2 Supplemental Information 2 Raw Data.

## Additional Information and Declarations

### Data Availability

The following information was supplied regarding data availability: The raw data are available in the Supplemental File. The World Clim data is available at: https://www.worldclim.org/data/worldclim21.html.
